# Stay Home, Stay Safe? The Impact of the COVID-19 Restrictions on the Prevalence, Nature, and Type of Reporter of Domestic Violence in the Netherlands

**DOI:** 10.1007/s10896-022-00473-8

**Published:** 2022-11-26

**Authors:** Anne Coomans, David Kühling-Romero, Sjoukje van Deuren, Meintje van Dijk, Steve van de Weijer, Arjan Blokland, Veroni Eichelsheim

**Affiliations:** 1 Netherlands Institute for the Study of Crime and Law Enforcement (NSCR), Amsterdam, Netherlands; 2 Department of Criminology, Faculty of Law, Vrije Universiteit Amsterdam, Amsterdam, Netherlands; 3 Department of Criminal Law and Criminology, Faculty of Law, Leiden University, Leiden, Netherlands; 4 Department of Developmental Psychology, Faculty of Behavioural and Social Sciences, University of Groningen, Groningen, Netherlands

**Keywords:** COVID-19, Pandemic, Domestic violence, Child maltreatment, Intimate partner violence, Violence against parents, Elderly abuse, Reporters

## Abstract

**Purpose:**

Insecurities and social isolation resulting from the COVID-19 restrictions, may have elevated tensions at home, consequently increasing the risk of domestic violence. The present study aims to examine changes in the prevalence, nature, and type of reporter of domestic violence following the various restrictions implemented to control the spread of the COVID-19 virus in the Netherlands.

**Methods:**

All official domestic violence reports recorded by the 26 Dutch domestic violence agencies in 2019 and 2020 were collected and analyzed. Time-series forecasting analyses, using a SARIMAX model, were conducted to predict the trend of domestic violence reports during the first lockdown and to compare the predicted trend with the observed trend.

**Results:**

The observed trend of the registered prevalence of domestic violence did not substantially differ from the predicted trend based on pre-pandemic data. Similarly, findings regarding the nature of domestic violence suggest no clear divergence of pre-pandemic trends during the lockdown period. Nonetheless, a shift was found from professional reporters (e.g., the police) to non-professional reporters (e.g., neighbors).

**Conclusions:**

The prevalence of domestic violence reports in the Netherlands did not increase. However, the COVID-19 restrictions may have led citizens, especially neighbors, to detect domestic violence more often.

## Introduction

Domestic violence is a prevalent and serious social problem. The World Health Organization (WHO) estimates that nearly one-third of women have experienced some form of intimate partner violence (IPV), and up to a billion children have been subjected to any form of child maltreatment, involving physical, sexual, and psychological abuse (WHO, 2020, 2021). Domestic violence also constitutes a global health problem, as it can result in serious health consequences, ranging from post-traumatic stress to death (Bouillon-Minois et al., 2020). Limiting itself to violence between those aged 16 or over who are, or have been, intimate partners or family members, the British Home Office estimates that the costs of domestic violence in terms of physical and emotional harm, lost output, and health service use, to be over 34,000 British pounds per victim (Oliver et al., 2019). The current study focuses on officially reported incidents of domestic violence in the Netherlands. Domestic violence is used as a generic term for violence perpetrated by someone within the domestic circle of the victims, hence including IPV and child maltreatment, as well as violence against parents and elderly abuse (Mellaard & Meijl, 2021).

The COVID-19 pandemic has raised global and national concerns about its potential for increasing rates of domestic violence. Existing risk factors for domestic violence perpetration and victimization such as substance abuse, the accumulation of stressful events in life, a lack of perceived support, and economic stressors are potentially amplified during times of crises such as disasters or a pandemic (Bell & Folkerth, 2016; Jenkins & Phillips, 2008). Many studies have found evidence for increasing rates of domestic violence after catastrophic events, often extending for several months or years (Gearhart et al., 2018; Jenkins & Phillips, 2008; Lauve-Moon & Ferreira, 2017).

Globally, restrictions such as lockdowns, curfews, and social distancing measures have been implemented to reduce the spread of the COVID-19 virus. Similar to the reported changes experienced after disasters, these restrictions have led to rapidly increasing stress and sudden shifts in daily routines (Campbell, 2020; Dong & Bouey, 2020). In the longer term, these restrictions may also have financial or economic consequences that contribute to heightened stress levels (Douglas et al., 2020). In the Netherlands, the government imposed the first lockdown between March 23, 2020, and May 1, 2020, in which people were asked to keep their distance, stay at home, and work from home as much as possible. In addition, schools, day-care centers, and many businesses – like restaurants, bars, and nightclubs—were closed completely. The lockdown limited citizens’ freedom of movement and increased levels of social isolation (Douglas et al., 2020). Suddenly, partners, families, and parents with children were forced to spend time only with each other. For some, these restrictions may have reduced stress as a result of fewer obligations and more time spent with relatives. For others, especially the more vulnerable groups in society living in small houses (e.g., individuals with a migration background or low social economic status), these restrictions might have created or increased tensions between household members. If not for everyone, then at least for some, the lockdown restrictions introduced a stressor that, given its impact and nature, could be expected to increase the likelihood of domestic violence during and perhaps even beyond the period of restrictions.

Similar to the period following disasters, lockdown restrictions may have created difficulties for victims of domestic violence in seeking help, while also limiting the possibilities for professionals to detect domestic violence and provide support (Bergman et al., 2021; Bradbury-Jones & Isham, 2020). The loss of social support and social contacts in the aftermath of a disaster is assumed to be an important factor in increased domestic violence during such periods. This is in line with the *social support deterioration model*, tested and introduced by Kaniasty and Norris (1993). This model posits that social networks tend to be disrupted after catastrophic events and resources of community support can become depleted when the needs of disaster victims exceeds the availability of resources. The disruption of social networks in combination with low expectations of available support is found to be associated with greater distress among victims of disasters (Kaniasty & Norris, 1995; Spencer et al., 2021).

Although the COVID-19 pandemic had no physically destructive impacts on infrastructures, access to domestic violence services has been limited during lockdown periods. Assistance from such services suddenly had to be provided remotely. Domestic violence shelters were unable to operate at capacity due to physical distancing and staff shortages related to caregiving responsibilities for self or others with COVID-19 infections (Campbell, 2020; Sharma & Borah, 2020). Combined, these circumstances may have posed a particular risk for already vulnerable families or individuals not yet known to authorities. Similar to disasters, albeit in different circumstances, social deterioration may have occurred as a result of the restrictions. During a lockdown period, social isolation and lack of perceived support may have increased feelings of stress causing problems in families to escalate, while at the same time these problems were likely to become less visible to the outside world (Anderberg et al., 2020).

In addition, the restrictions may have differentially altered the risk of victimization of domestic violence due to home confinement. As domestic violence usually occurs behind closed doors (Bradbury-Jones & Isham, 2020), it is conceivable that, as a result of the stay-at-home orders, some types of domestic violence (such as IPV and child maltreatment) were more likely to increase than others (such as elderly abuse and violence between siblings living separately from one another).

Finally, the restrictions may have affected the likelihood that domestic violence was reported to authorities. On the one hand, professionals might be less likely to identify incidents. On the other, these same restrictions could have made neighbors, community members, and other bystanders more likely to detect domestic violence and report their concerns to authorities. In the Netherlands, the government launched a national campaign ‘Domestic Violence During the COVID-19 Pandemic’, specifically aimed at encouraging bystanders to report suspicions of domestic violence to authorities. Subsequently, Dutch citizens could have become more aware and more willing to report domestic violence to the official domestic violence agencies.

### Domestic Violence during COVID-19 Pandemic

As the pandemic continues, the reported impacts of the implemented restrictions on domestic violence rates are mixed (Bergman et al., 2021). Two recent systematic reviews provide an overview of the studies that investigated the impact of the COVID-19 pandemic. Piquero et al. (2021) included studies that had a measurable and codable domestic violence outcome based on official records that were assessed both prior to and post restrictions to control the COVID-19 virus, thereby excluding retrospective self-reports and literature reviews. Only 18 out of the 132 initially selected articles could be included. The time span of available data of the eligible studies was relatively short, ranging from several weeks to several months between the pre-and post-COVID-19 related restrictions. A total of 37 change estimates of COVID-19 restrictions on domestic violence were distilled from these 18 studies, with 29 effect estimates indicating a significant increase in domestic violence rates after the first implemented lockdown, and the other eight effect estimates implying a significant decline in domestic violence rates. The meta-analysis resulted in an average rise in domestic violence rates after the COVID-19 pandemic of 7.86%.

In the other systematic review of Kourti et al. (2021), case reports, cohort studies, cross-sectional studies, case series, and case–control studies were included, as well as data from domestic violence organizations, human rights organizations, and the police. Out of 77 eligible studies, 32 studies were included. Only four studies overlapped in both systematic reviews, possibly as a result of the different inclusion criteria (Leslie & Wilson, 2020; McLay, 2021; Mohler et al., 2020; Piquero et al., 2020). Whereas most included studies found an increase in domestic violence rates during the first week of the first lockdown, these rates seemed to decline as the pandemic continued. Furthermore, discrepancies were found between official domestic violence records of the police and helpline calls; while official domestic violence reports decreased, helpline calls appeared to increase. Both types of data are susceptible to bias (Kourti et al., 2021), and different issues may be reported in helplines calls than to the police, for instance, due to personal barriers such as fear or distrust (Wolf et al., 2003). Considering these findings, the authors of this review concluded that it is still impossible to make unambiguous statements about the impact of the COVID-19 pandemic on domestic violence (Kourti et al., 2021).

Several gaps arose from the literature. First, the time span of the data on which some of these studies rely was relatively short. For example, some studies used data from January 1^st^, 2020 (or any time later) until just several weeks into the beginning of the COVID-19 pandemic (Hsu & Henke, 2021; Leslie & Wilson, 2020; Mohler et al., 2020; Piquero et al., 2020). Due to long-term trends and seasonal influences (e.g., holidays), it is important to include wider timespans to get a better understanding of possible changes in domestic violence rates. For example, Piquero et al. (2020) used data from the Dallas Police Department to investigate whether the stay-at-home orders were associated with a shifting pattern in the prevalence of domestic violence using a time series forecasting model. The authors found an increase in domestic violence in the two weeks right after the implementation of the stay-at-home orders, but a decrease in domestic violence thereafter. The initially higher rates of domestic violence, however, could not directly be attributed to the stay-at-home orders since there had been already an upward pattern of domestic violence rates sometime prior to the implemented COVID-19 restrictions.

Another gap in the existing research is that many studies focus either solely on IPV or solely on child maltreatment, whereas other studies rely upon domestic violence police data, in which clarification about whether domestic violence refers to IPV or child maltreatment is often missing (Kourti et al., 2021). Furthermore, potential changes in other types of domestic violence during the COVID-19 pandemic, such as violence against parents, and elderly abuse are currently understudied. It could be expected that the restrictions during periods of lockdown altered the occurrence of some types of domestic violence, but not others: violence between family members living together may occur more frequently than violence between other members of a family, as those members are less likely to be confined in a home together.

Finally, little is known about potential changes in the *reporters* of domestic violence during the COVID-19 pandemic. Many professionals who typically detect domestic violence suddenly had to work from home during the first lockdown. Especially during this first lockdown, only a few professions were classified as essential (e.g., healthcare, police, ambulance), and only children from parents working in these essential sectors were allowed to be in day-care. Domestic violence shelters remained open, but with strict restrictions regarding the in and outflow of residents. In addition, research has shown that 1 in 5 Dutch individuals avoided healthcare during the first lockdown, often while experiencing potentially urgent symptoms (Splinter et al., 2021). Due to the reduced contact between professionals and potential victims, it is possible that professionals detected domestic violence among their clientele less often and consequently made fewer reports to the authorities, contributing to declining rates of domestic violence reports. At the same time, the role of other home-confined citizens, such as neighbors, in detecting and reporting domestic violence may have increased. The findings of Bullinger et al. (2021) confirm that interactions between civilians and police officers declined as a result of the social distance measure. Furthermore, while the stay-at-home orders led to increased domestic violence calls for police (i.e., 911 calls), levels of reported domestic violence in more formal channels (i.e., official reported crime and police arrests for domestic violence) decreased. These findings may mask important variations in domestic violence-related reporting across households as the decline in official reported domestic violence was driven by households living in houses rather than apartments. Hence, proximity seems to be an important factor in reporting domestic violence (Bullinger et al., 2021). However, as information about the reporters is often unknown, the role of reporters of domestic violence remains unclear (Bullinger et al., 2021; Ertan et al., 2020; Sharma & Borah, 2020).

### Current Study

This study adds to the existing literature on domestic violence during the COVID-19 pandemic in at least three different ways. First, the study relies upon official domestic violence *records* starting prior to the introduction of the first restrictions to control the COVID-19 virus (January 1^st^, 2019) until the period after the strictest control measures were scaled down (December 13^th^, 2020). This allows for taking long-term trends and seasonal influences into account. Using time-series forecasting models, we ascertain the extent to which the reported prevalence of domestic violence during the pandemic deviates from what could be expected based on pre-pandemic data. Second, instead of focusing solely on one type of domestic violence, this study addresses domestic violence by distinguishing between different types of violence, including violence against parents and elderly abuse. Third, the assumption that bystanders may play a vital role in detecting domestic violence during strict lockdown periods has, to our knowledge, not yet been properly tested. For this study we have data on who reported domestic violence, allowing us to examine possible changes in the type of reporter during the pandemic. In sum, this study attempts to answer the following research question: Are there any observed changes in the prevalence, nature, and type of reporter of domestic violence as a result of the various implemented COVID-19 restrictions in the Netherlands?

## Data and Methods

### Data

The present study used data from *Veilig Thuis* (Safe Home), the official domestic violence agency in the Netherlands. Upon contact with a reporter of domestic violence, employees of Veilig Thuis evaluate the reported situation regarding the safety of all those involved, which can include victims, perpetrators, or bystanders who witness the domestic violence situation, through a structured and validated safety assessment. Based on this initial assessment, the agency either decides to refer to specialized care or to start a more elaborate investigation. Depending on the investigation’s findings, the agency may initiate potentially more intrusive interventions by, for example, alerting the child protection agency or assisting victims in filing an official complaint against their aggressor to the police. Regardless of this evaluation, all reported situations are recorded by the agencies. The data constitute all daily domestic violence reports made in all 26 regions between January 1^st^, 2019 and December 13^th^, 2020, and include information on the number of daily reports (*N* = 246,688), the number of unique domestic violence cases (*N* = 190,324) underlying these reports, the presumptive nature of domestic violence, and the reporters of domestic violence.^1^ In the remainder of this article, when we discuss domestic violence in our data, we only refer to *officially reported incidents* of domestic violence. A distinction was made between domestic violence *cases* and *reports* given the possibility that multiple people can report the same or repeated incident(s) of suspected domestic violence, which eventually results in the registration of one unique case. Therefore, in our calculations of the prevalence of domestic violence cases, only the first time a unique case was reported was analyzed. Regarding the prevalence of domestic violence reports, all reports were analyzed.

Table 1 provides the descriptive statistics of the variables in our study. Multiple forms of domestic violence can occur simultaneously in one case (e.g., IPV and child maltreatment). When multiple forms of domestic violence were registered within the same case, we recoded it into one category by letting one category prevail above another in the respective order as presented in Table 1, based on the extent to which the different types of domestic violence actually occur (except for other problems, since this is a residual category)*.* Regarding reporters of domestic violence, the domestic violence agency keeps track of the origin of a professional report and the role of non-professional reporters.

**Table 1:** Descriptive statistics of the variables of analysis

Variable	Categories
Presumptive nature of domestic violence	1. IPV and child maltreatment^a^ 2. Child maltreatment 3. IPV 4. Violence against parents 5. Violence against the elderly 6. Other problems^b^
Reporter of domestic violence	1. Professional reporter 2. Non-professional reporter
Function of professional reporter	1. Justice and Safety (i.e., in most cases the police) 2. (Para)medical professions (e.g., general practitioners) 3. Psychologists, social workers and psychotherapists 4. Education and day-care 5. Other professionals
Role of non-professional reporter	1. Directly involved children 2. Directly involved adults 3. Family members of those directly involved 4. Persons belonging to the social network of those directly involved 5. Neighbors 6. Other non-professional reporters, including volunteers^c^

^a^It should be noted that a case is (also) classified as child maltreatment when children are witnesses of violence between their parents.

^b^Other problems include ‘other domestic violence’ (violence committed by someone from the victim’s family circle, in which domestic violence refers to the relationship between perpetrator and victim; for example, violence between brother and sister) and ‘problems other than domestic violence’.

^c^Non-professional reporters, such as family members or neighbors, can make an anonymous report of domestic violence. Information about the role of a non-professional reporter is not registered when a person reports a suspicion of domestic violence anonymously resulting in a missing value.

The domestic violence agency changed its registration policy as of January 1^st^, 2019. Accordingly, it was not possible to collect the domestic violence reports prior to 2019 as each domestic violence agency had its own policy on registering the reports. Since the policy change, domestic violence reports are registered more systematically. However, because of the implemented policy change and especially during the first quarter of 2019, detailed information about the presumptive nature and type of reporter of domestic violence was sometimes missing as a result of the policy change being implemented.

### COVID-19 Restrictions in the Netherlands

On March 11^th^, 2020, the WHO declared COVID-19 as a pandemic. The Dutch government introduced the first restrictions on March 15^th^, 2020, which were aimed at staying-at-home, social distancing, and the closure of restaurants, bars, cafés, sports locations, museums, schools, and day-care centers. Between March 15^th^, 2020, and May 11^th^, 2020, new restrictions were introduced (such as the complete closure of long-term care facilities), some restrictions were tightened (for example, all events and social gatherings were banned), and most existing restrictions were extended. On May 11^th^, 2020, some restrictions were gradually relaxed. Primary schools and day-care centers (partially) reopened first. On June 1^st^, 2020, secondary schools reopened again, after which high schools and universities reopened on June 16^th^, 2020. Around October 2020, infection rates seemed to increase again rapidly, urging the Dutch government to implement restrictions again. On October 13^th^, 2020, a partial lockdown was declared in which individuals could meet up with a maximum of three persons per day, events and social gatherings were banned, and restaurants, cafés, and bars had to shut their doors again. Based on the up- and downscaling of the COVID-19 restrictions in 2020, we distinguished four different ‘COVID-19 periods’: no restrictions (January, 1^st^), first lockdown (March, 15^th^), relaxation restrictions (May, 11^th^), and partial lockdown (October, 13^th^). Based on the start dates of these four periods, we identified the weeks in our data for 2019 and 2020.^2^

### Analytic Strategy

#### Bivariate Analyses

Bivariate analyses were conducted to examine possible changes in domestic violence cases and reports, the nature of domestic violence, and the type of reporter. The records in 2019 were used as a benchmark, to which the records in 2020 were compared across the four identified COVID-19 periods. For the bivariate analyses, Chi-square tests were performed with additional Bonferroni post-hoc tests.

#### Time Series Forecasting Model

Based on the daily number of official domestic violence records prior to the first lockdown (January 1^st^, 2019 – March 15^th^, 2020), we used Seasonal Auto-Regressive Integrated Moving Average with eXogenous variables (SARIMAX) models to predict domestic violence trends during the 80 days of the first lockdown, starting from March 16^th^, 2020. As these predicted trends are based on data prior to the pandemic, these trends are assumed to reflect the counterfactual situation i.e., if the COVID-19 virus had never occurred. In addition, we chose to rely upon daily counts of the data, because domestic violence reports depend on people’s daily routines. The data, for example, showed that the number of reports was consistently lowest during the weekends and official holidays and the highest on the days after holidays and on Mondays. As such daily routines may have changed because of the COVID-19 restrictions, we were interested to capture daily trends.^3^ Various time series were constructed for the total amount of domestic violence cases per day, the number of cases per day for each type of domestic violence, and the number of reports per day for each type of reporter, now referred to as domestic violence trends. The models generated a predicted number of domestic violence trends and a 95% confidence interval, indicating the range in which the model expects the number of domestic violence cases would fall in 95% of the time. If the observed number of domestic violence trends fell outside the upper or lower bounds of the confidence intervals on many instances, substantial differences were observed between the expected and observed number of domestic violence trends. A total of nine dummy variables were entered into the SARIMAX models to consider structural weekly trends observed in the data (e.g., a high number of reports on Mondays, low numbers of reports during weekends), national Dutch holidays, and the day after holidays (as rebound effects of reporting were observed during these days). The parameters for the SARIMAX models were automatically determined by applying the Hyndman – Khandakar algorithm in the fpp3 package in R (Hyndman & Athanasopoulos, 2021; Hyndman & Khandakar, 2008). Additional information on the SARIMAX models is provided in the Appendix.

A longer forecasting period of 276 days (i.e., between March 15^th^ – December 13^th^, 2020) was also applied using the data prior to the first lockdown, to examine a potential delay in reported domestic violence cases due to the implementation of the COVID-19 restrictions. A delay could be expected because, for instance, domestic violence cases were not reported immediately to the relevant agencies during the first lockdown. As schools and day-care centers, for example, were completely closed during this period, cases of domestic violence may have remained hidden. With the reopening of schools and day-care centers, professionals could have observed certain signs of domestic violence among their apprentices that perhaps occurred during the lockdown period.

## Results

**Fig. 1:**
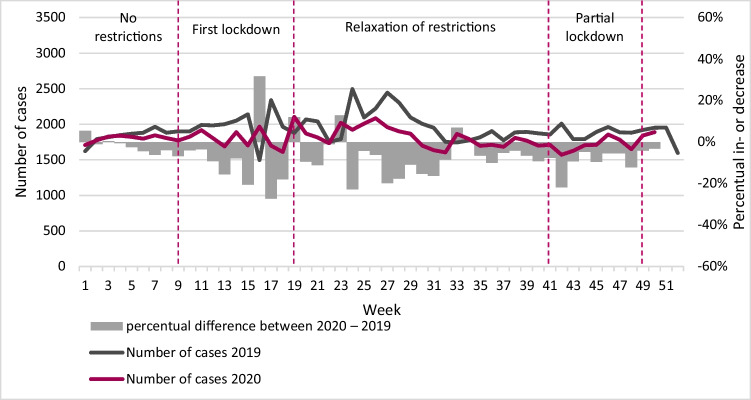
Absolute number of cases and year-over-year percent change in weekly cases. Note: 2019: *N* = 100,460; 2020: *N* = 89,864 (up until week 50)

**Fig. 2:**
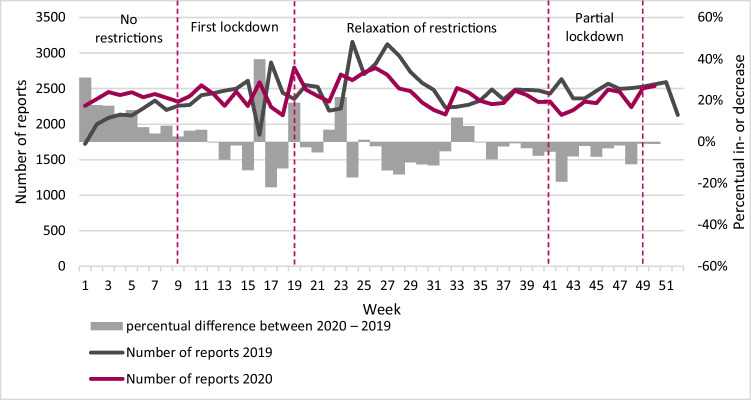
Absolute number of reports and year-over-year percent change in weekly reports. Note: 2019: *N* = 126,339; 2020: *N* = 120,349 (up until week 50)

Figure 1 presents the prevalence of domestic violence *cases* (reflecting the trend in new domestic violence cases), while Fig. 2 presents the prevalence of domestic violence *reports*. As mentioned before, this distinction was made as it is possible that multiple reports were filed about the same domestic violence case. Overall, the number of cases of domestic violence was smaller in 2020 compared to 2019 (Fig. 1). A similar pattern is observed in the number of reports, except for the period before the first lockdown (Fig. 2). During this period, the number of reports was larger compared to the same period in 2019. However, from the first lockdown onwards, the number of reports was smaller compared to the same periods in 2019. Combining information on cases and reports, we explored whether the number of reports on a single case of domestic violence differed following the COVID-19 restrictions. As shown in Table 2, the maximum and the average number of reports per case were approximately the same across the different (COVID-19) periods in 2019 and 2020.^4^

**Table 2:** Minimum, maximum and average number of reports per case, per COVID-19 period, per year

	No restrictions (Week 1–9)				First lockdown (Week 10–18)				Relaxation of restrictions (Week 19–41)				Partial lockdown (Week 42–49)			
	N	Min	Max	Mean	N	Min	Max	Mean	N	Min	Max	Mean	N	Min	Max	Mean
2019	16,597	1	9	1.15 (.46)	18,764	1	12	1.16 (.50)	47,475	1	11	1.23 (.62)	17,060	1	9	1.17 (.50)
2020	18,274	1	8	1.17 (.50)	18,177	1	11	1.17 (.50)	45,439	1	13	1.24 (.65)	15,686	1	9	1.19 (.53)

Table 3 shows the proportion of cases in which the different types of domestic violence presumably occurred. Over all periods, the proportion of cases in which IPV and/or child maltreatment was reported significantly increased between 2019 and 2020, while the proportion of cases in which other problems (types of violence that cannot be classified under the other categories or other problems than domestic violence) occurred significantly decreased in 2020 compared to the same periods in 2019. During the first lockdown, a significant increase was found in the proportion of cases in which violence against parents and elderly abuse was reported, compared to the same period in 2019. During the relaxation of restrictions, a significant increase was found in the proportion of cases in which violence against parents was reported as well. Note that (changes in) the percentages of different types of domestic violence could be biased when the missing values on this variable are not equally distributed across the different types for the subsequent periods under study. This also applies to the findings presented in Tables 4–6.

**Table 3:** Proportion of the presumptive nature of domestic violence in cases, per COVID-19 period, per year

	No restrictions (Week 1–9)			First lockdown (Week 10–18)			Relaxation of restrictions (Week 19–41)			Partial lockdown (Week 42–49)		
	2019 (%)	2020 (%)	Sig	2019 (%)	2020 (%)	Sig	2019 (%)	2020 (%)	Sig	2019 (%)	2020 (%)	Sig
IPV and child maltreatment	11.9	16.0	*	13.6	17.8	*	16.0	18.5	*	15.8	19.2	*
Child maltreatment	28.1	29.7	*	30.9	31.2		31.0	32.8	*	31.6	33.5	*
IPV	20.0	20.6		18.0	19.9	*	18.6	19.8	*	18.9	18.4	
Violence against parents (under 65 years)	3.3	3.2		2.9	3.5	*	2.9	3.4	*	3.1	3.0	
Elderly abuse (above 65 years)	1.7	1.9		1.7	2.0	*	1.9	1.9		1.7	1.8	
Other problems	35.0	28.6	*	33.0	25.7	*	29.7	23.5	*	28.9	24.1	*
Total	100	100		100	100		100	100		100	100	

* Shows results of Bonferroni post-hoc tests whether the different forms of domestic violence differ significantly between 2019 and 2020 at *p* < .05

Missings in 2019: weeks 1–9: 11.2% (*N* = 1,850); weeks 10–18: 4.2% (*N* = 755); weeks 19–41: 3,0% (*N* = 1,342); weeks 42–49: 2.8% (*N* = 418)

Missings in 2020: weeks 1–9: 2.3% (*N* = 375); weeks 10–18: 1.7% (*N* = 269); weeks 19–41: 1.6% (*N* = 657); weeks 42–49: 4.5% (*N* = 615)

**Table 4:** Proportion of professional and non-professional reporters, per COVID-19 period, per year

	No restrictions (Week 1–9)			First lockdown (Week 10–18)			Relaxation of restrictions (Week 19–41)			Partial lockdown (Week 42–49)		
	2019 (%)	2020 (%)	Sig	2019 (%)	2020 (%)	Sig	2019 (%)	2020 (%)	Sig	2019 (%)	2020 (%)	Sig
Professional reporters	93.0	92.4	*	92.5	90.7	*	91.1	88.9	*	92.2	90.8	*
Non-professional reporters	7.0	7.6	*	7.5	9.3	*	8.9	11.1	*	7.8	9.2	*
Total	100	100		100	100		100	100		100	100	

* Shows results of Bonferroni post-hoc tests whether the type of reporter differ significantly between 2019 and 2020 at *p* < .05

Missings in 2019: weeks 1–9: 7.5% (*N* = 1,429); weeks 10–18: 1.0% (*N* = 218); weeks 19–41: 0.7% (*N* = 420); weeks 42–49: 0.3% (*N* = 62)

Missings in 2020: weeks 1–9: 0.3% (*N* = 58); weeks 10–18: 0.2% (*N* = 50); weeks 19–41: 0.3% (*N* = 157); weeks 42–49: 0.3% (*N* = 48)

**Table 5:** Proportion of different professions of professional reporters, per COVID-19 period, per year

	No restrictions (Week 1–9)			First lockdown (Week 10–18)			Relaxation of restrictions (Week 19–41)			Partial lockdown (Week 42–49)		
	2019 (%)	2020 (%)	Sig	2019 (%)	2020 (%)	Sig	2019 (%)	2020 (%)	Sig	2019 (%)	2020 (%)	Sig
Justice and Safety	73.8	73.7		73.1	74.9	*	75.3	74.8	*	72.3	71.9	
(Para)medical professions	8.7	8.8		8.6	7.8	*	8.5	8.9	*	8.9	8.4	
Psychologists, social workers, and psychotherapists	7.1	7.9	*	8.3	8.5		7.9	7.7		8.6	8.3	
Education and day-care	4.4	4.6		4.6	3.3	*	3.8	3.5		5.2	5.5	
Other professionals	6.0	4.9	*	5.3	5.5		4.5	5.0	*	5.1	5.9	*
Total	100	100		100	100		100	100		100	100	

* Shows results of Bonferroni post-hoc tests whether the professions of professional reporters differ significantly between 2019 and 2020 at *p* < .05.

Missings in 2019: weeks 1-9: 5.9% (*N* = 972); weeks 10-18: 6.0% (*N* = 1,203); weeks 19-41: 4.0% (*N* = 2,109); weeks 42-49: 4.0% (*N* = 727).

Missings in 2020: weeks 1-9: 4.0% (*N* = 793); weeks 10-18: 1.5% (*N* = 284); weeks 19-41: 0.3% (*N* = 174); weeks 42-49: 0.2% (*N* = 34).

**Table 6:** Proportion of different groups of non-professional reporters, per COVID-19 period, per year

	No restrictions (Week 1–9)			First lockdown (Week 10–18)			Relaxation of restrictions (Week 19–41)			Partial lockdown (Week 42–49)		
	2019 (%)	2020 (%)	Sig	2019 (%)	2020 (%)	Sig	2019 (%)	2020 (%)	Sig	2019 (%)	2020 (%)	Sig
Directly involved, youth	3.1	3.5		4.2	2.9	*	2.4	2.4		3.8	2.8	
Directly involved, adults	25.9	27.8		24.0	26.5		21.3	22.1		23.9	24.0	
Family members	20.1	20.7		18.3	16.0		17.1	14.9	*	18.5	18.3	
Persons belonging to the social network	11.9	10.4		11.7	9.1	*	9.1	8.7		10.9	9.7	
Neighbors	21.5	21.3		24.6	29.3	*	30.1	30.2		21.8	23.8	
Other citizens	17.5	16.3		17.1	16.2		20.0	21.8	*	21.0	21.5	
Total	100	100		100	100		100	100		100	100	

* Shows results of Bonferroni post-hoc tests whether the roles of non-professional reporters differ significantly between 2019 and 2020 at *p* < .05.

Missings in 2019: weeks 1-9: 5.2% (*N* = 73); weeks 10-18: 5.3% (*N* = 87); weeks 19-41: 4.5% (*N* = 223); weeks 42-49: 2.7% (*N* = 47).

Missings in 2020: weeks 1-9: 2.3% (*N* = 41); weeks 10-18: 2.0% (*N* = 43); weeks 19-41: 2.3% (*N* = 137); weeks 42-49: 1.6% (*N* = 31).

The proportion of reports coming from professional and non-professional reporters is shown in Table 4. Although the largest proportion of reports were reported by professional reporters, a significant shift was found from professional to non-professional reporters between the different periods in 2020 as compared to 2019. During the first lockdown, the proportion of reports reported by professionals significantly decreased, while the proportion of non-professional reports significantly increased (e.g., during the first lockdown: 9.8% in 2020 versus 7.6% in 2019).

The proportion of the different professions in which the professional reporters were employed is shown in Table 5. Small differences were found between the different COVID-19 periods, compared to the same periods in 2019. During the first lockdown, the proportion of reports reported by professionals working in the sector Justice and Safety (mainly the police) significantly increased (75.8% in 2020 versus 73.5% in 2019). The proportion of reports reported by from professionals working in day-care and education (2.9% in 2020 versus 4.6% in 2019), and (para)medical professions (7.8% in 2020 versus 8.5% in 2019) significantly decreased during the first lockdown, compared to the same period in 2019.

Differences are visible in the proportion of reports reported by different groups of non-professional reporters, see Table 6. The proportion of reports reported by neighbors significantly increased during the first lockdown (30.9% in 2020 versus 25.1% in 2019). On the contrary, a significant decline was found in the proportion of reports reported by directly involved youth, family members, and persons belonging to the social network during the first lockdown, as compared to 2019. Finally, by breaking down the number of reports for each case by professional and non-professional reporters, we found that these are highly similar across both years.

### SARIMAX Forecasting Model

#### Prevalence of Domestic Violence

**Fig. 3:**
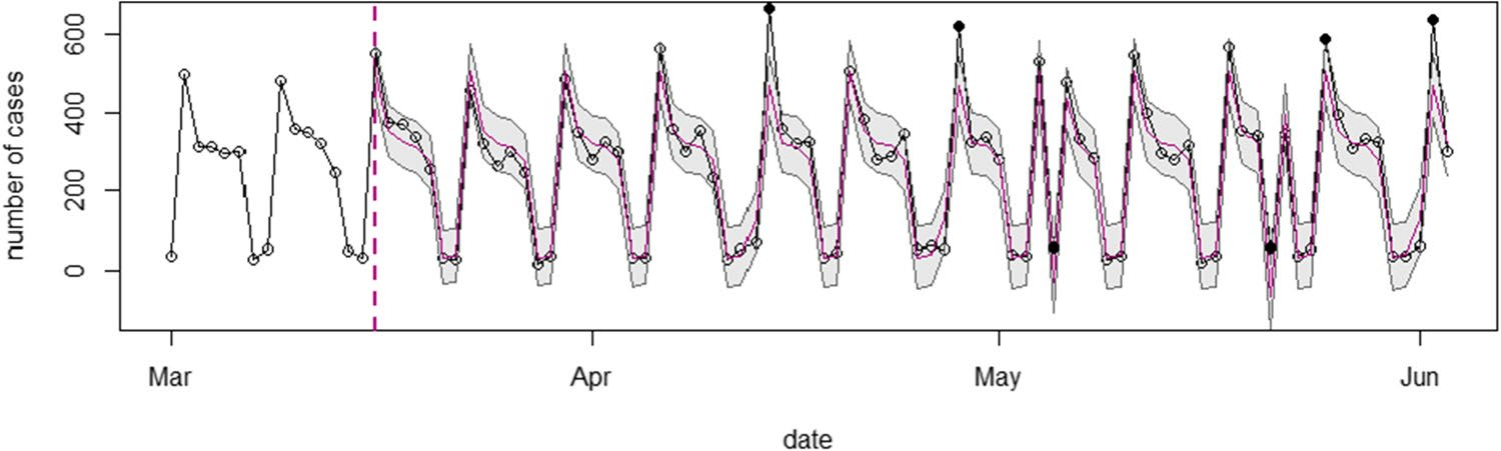
SARIMAX model of the observed and expected number of domestic violence cases during the first lockdown. Note: The x-axis represents the number of forecasted days into the first lockdown. The starting date is Sunday March 1st, 2020.The y-axis shows the number of reported and predicted domestic violence cases. Inside the graph, the black line represents the actual number of domestic violence cases per day, the red line is the mean daily prediction, and the confidence interval of the predictions is represented by the blue area around this line. The black dots represent the observed data outside of the prediction confidence interval

**Fig. 4:**
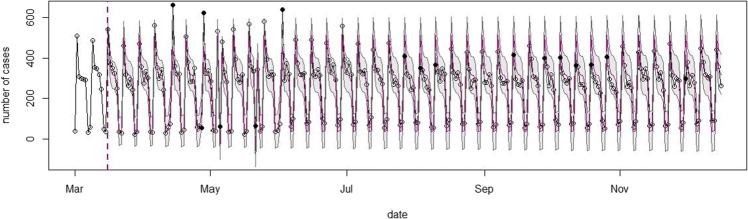
Extended SARIMAX model of the observed and expected number of domestic violence cases during the COVID-19 pandemic. Note: The x-axis represents the number of forecasted days into the COVID-19 pandemic The starting date is Sunday March 1st, 2020. The y-axis shows the number of reported and predicted domestic violence cases. Inside the graph, the black line represents the actual number of domestic violence cases per day, the red line is the mean daily prediction, and the confidence interval of the predictions is represented by the blue area around this line. The black dots represent the observed data outside of the prediction confidence interval

Figure 3 presents the forecasted prevalence of domestic violence cases during the 80 days of the first lockdown period (*N* = 21,094). The Figure shows that the number of reported domestic violence cases fell outside the confidence intervals of the predictions on six out of 80 days, indicated by the black dots, further referred to as mismatches. On four of these days, the observed number of cases surpassed the predictions. On the other two days, the prediction exceeded the observed number of cases. All mismatches, however, fell on a holiday or the day after a holiday. The forecasted model over a longer period (*N* = 71,153), as presented in Fig. 4, reveal seventeen mismatches, especially on Mondays, in almost six consecutive weeks from the 14^th^ of September 2020 onwards. On these Mondays, the observed number of cases was lower than what was predicted by the model. Overall, these findings indicate that the observed number of domestic violence cases during, but also for some weeks after the first lockdown, did not differ substantially from the expected number of domestic violence cases. If anything, cases appeared lower than what could be expected in the absence of the COVID-19 restrictions at the end of 2020.

#### Nature of Domestic Violence

The SARIMAX models differentiated by the nature of domestic violence cases show similar results as described for the prevalence of domestic violence cases (see Table 7). For ‘IPV and child maltreatment’, nine mismatches (out of 80 days) occurred between the predicted and observed data. Four of these mismatches fell on a day after a holiday. Fewer mismatches were observed for all other types of domestic violence, with some of them relating to holidays or days immediately after. These findings indicate that the predicted and observed number of the different types of domestic violence do not substantially differ from each other.

**Table 7:** Plots of daily SARIMAX models for nature of domestic violence during the first lockdown

	Mismatches	Overestimations	Underestimations	Holiday	Day after holiday
IPV and child maltreatment	9	0	9	0	4
Child maltreatment	5	0	5	0	3
IPV	3	0	3	0	2
Violence against parents	4	0	4	0	1
Elderly abuse	3	1	2	0	1
Other problems	3	0	3	1	1

#### Type of Reporter of Domestic Violence

**Fig. 5:**
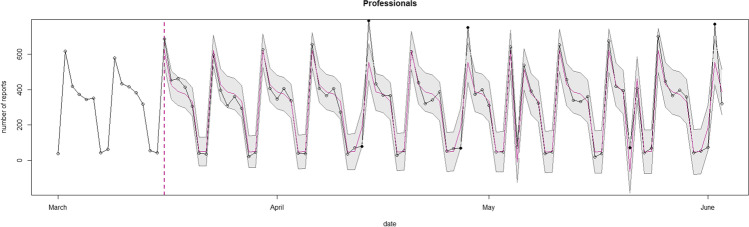
SARIMAX model of the observed and expected number of professional reports during the first lockdown. Note: The x-axis represents the number of forecasted days into the first lockdown. The starting date is Sunday March 1st, 2020. The y-axis shows the number of reported and predicted professional reports. Inside the graph, the black line represents the actual number of professional reports per day, the red line is the mean daily prediction, and the confidence interval of the predictions is represented by the blue area around this line. The black dots represent the observed data outside of the prediction confidence interval

**Fig. 6:**
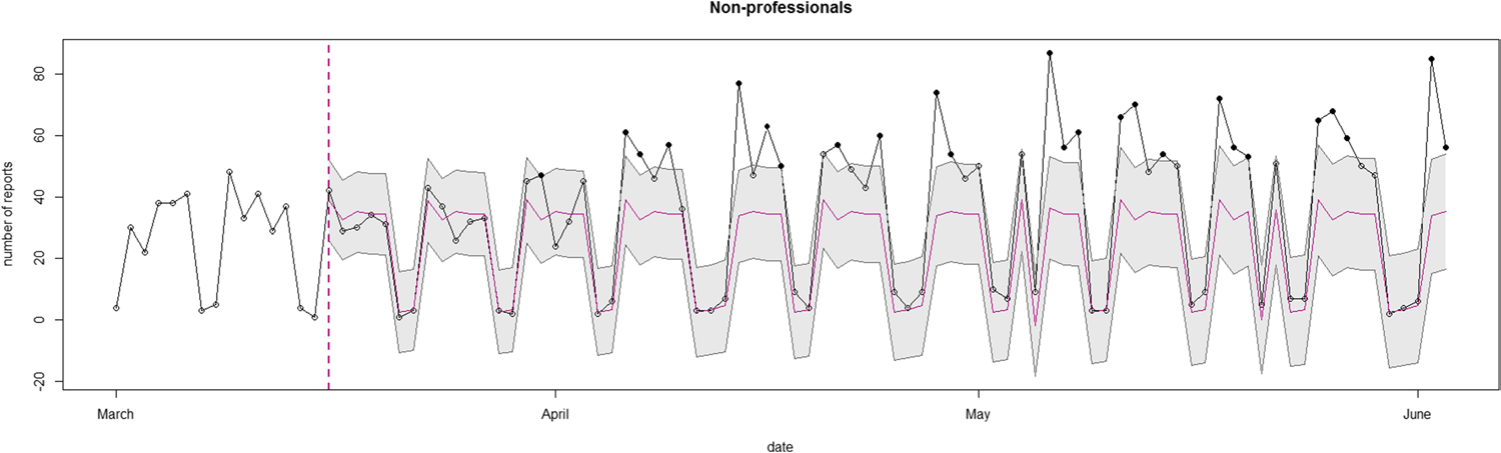
SARIMAX model of the observed and expected number of non-professional reports during the first lockdown. Note: The x-axis represents the number of forecasted days into the first lockdown. The starting date is Sunday March 1st, 2020. The y-axis shows the number of reported and predicted non-professional reports. Inside the graph, the black line represents the actual number of non-professional reports per day, the red line is the mean daily prediction, and the confidence interval of the predictions is represented by the blue area around this line. The black dots represent the observed data outside of the prediction confidence interval

Figure 5 and 6 show two SARIMAX models investigating the patterning of the number of reports made by professionals and non-professionals. Figure 5 shows six (out of 80) days on which the observed professional reports fell outside the confidence intervals. The observed number of professional reports was below the predictions in two days. In the remaining four days, the observed number of professional reports surpassed the predictions. All these mismatches fell on a holiday or the day after a holiday, meaning these (day after) holidays could explain the mismatches found.

Figure 6 presents the predicted and observed number of non-professional reports. On 25 (out of 80) days, the observed non-professional reports fell outside the confidence intervals. On these days, the predicted number of non-professional reports was lower than the observed number of non-professional reports. From the second week of April until the end of the first lockdown, every week contained two to four mismatches between the observed and predicted number of non-professional reports per day.

As our results suggest an overall similar pattern of domestic violence reports in 2020 as compared to 2019, one could expect that, if the number of non-professionals reports systematically exceeds the predicted trend, the observed number of professional reports should be below the expected trend more so than our findings show. However, we must bear in mind that the absolute number of professional reports is much higher than the absolute number of non-professional reports, and that therefore a relative increase in the number of non-professional reports does not readily translate into a marked relative decrease in the number of professional reports. Furthermore, as noted in Table 2, the type of reporter had a lot of missing values during the first ten weeks of 2019. As these missing values could affect the SARIMAX models, we also estimated the models excluding the first ten weeks of 2019. The conclusions based on these additional analyses did not differ from those presented here. Finally, we performed the SARIMAX models on weekly counts as a robustness check. Similar results could be drawn as for the SARIMAX models performed on daily counts (see Appendix).

## Discussion

The aim of this study was to examine possible changes in trends of domestic violence reports after the first restrictions to control the COVID-19 virus were introduced in the Netherlands. We used national data on domestic violence reports in 2019 and 2020 as recorded by the official domestic violence agency in the Netherlands, including information about the prevalence, nature, and type of reporter of domestic violence.

The findings of this study indicate that, in the Netherlands, the registered prevalence (i.e., trends) of domestic violence cases in 2020, as compared to 2019, did not increase after the first COVID-19 restrictions were introduced. In addition, the findings of our SARIMAX models – taking into account long-term trends and seasonality – show that the observed trend does not substantially differ from the expected trend of the registered domestic violence cases during the first lockdown.

These findings can be interpreted in several ways. A first explanation would be that, potentially, the feared increase in actual domestic violence rates following the COVID-19 restrictions is absent. The stable pattern in the trend of domestic violence cases could be a result of the possibility that some families, households, or partners are doing worse during the COVID-19 crisis (e.g., vulnerable families), while other families are in fact doing better. After all, the lockdown may have led to less stress for some people, because many (social) obligations disappeared or were at least temporarily postponed, and people could spend more time with their own families. This is in line with a recent study into specific COVID-19 risk factors for perpetrating IPV (Spencer et al., 2021), which found that factors related to lifestyle changes and isolation, such as working from home, and the amount of time interacting with friends/family, did not significantly predict IPV perpetration. This could indicate that individuals may react differently to the sudden lifestyle changes posed by the COVID-19 pandemic (Spencer et al., 2021).

On the other hand, an absence of an increase in domestic violence rates – especially when analyzing registered data – does not have to mean that domestic violence has actually remained similar. The presumably already large dark figure of domestic violence may have been aggravated further by the COVID-19 pandemic as the possibility exists that a growing part of victims might not have found their way to the official domestic violence agencies and received the help they needed (Gearhart et al., 2018). Under this scenario, the absence of change in the domestic violence reports may mask an increase in actual domestic violence incidents. Furthermore, the consequences of the COVID-19 pandemic may also only become visible after a longer period, such as the loss of economic ties and its associated stress, possibly delaying an increase in the prevalence of domestic violence. Nonetheless, the results of the longer SARIMAX model revealed that, at the end of 2020 when restrictions were again relaxed, the observed trend of the registered domestic violence cases was substantially lower than what would have been expected in the absence of COVID-19 restrictions.

We did not find a clear divergence of pre-pandemic trends for the presumptive nature of domestic violence as registered in the domestic violence reports. While bivariate analyses showed significant changes in the proportion of cases of certain types for each of the examined periods, the SARIMAX model found the observed daily values predominantly to be within the predicted trend.

Although only some of the professions in specific sectors (Ministry of Justice and Safety, healthcare, youth care, social support services, schools, day-care locations) in the Netherlands were considered ‘essential’ and hence remained open to the public, 1 in 5 Dutch citizens appeared to avoid healthcare during the first lockdown (Splinter et al., 2021). During the first lockdown, professionals nevertheless remained the most important reporters of domestic violence, partly because these professionals are obliged to report their suspicions to the official domestic violence agency. Still, we found a significant shift from professional to non-professional reporters during the first lockdown, indicating that the opportunities for both professional and non-professional reporters may have changed due to the COVID-19 restrictions (Nardi-Rodríguez & Paredes-López, 2022). By looking at the professional reporters in more detail, we found a decrease in the proportion of professional reports coming from education and day-care, and (para)medical professions. It has been argued that school and day-care closures may have been damaging for children in many respects (Hoffman & Miller, 2020). Our study seems to suggest that at least teachers and day-care workers had fewer opportunities to detect domestic violence in times of school closures. In times of new crises, it is important to stay alert to the negative consequences of the crises-related restrictions can have on children. In contrast to the findings of Bullinger et al. (2021), we found an increase in the proportion of reports reported by the police during the first lockdown. Although police reports are registered as professional reports by the domestic violence agency, one could argue that the initial reporters bringing suspicions of domestic violence to the attention of the police are often citizens, as the police mainly function as a reactive institution.

The SARIMAX models did provide evidence of a divergent trend in non-professional reporters after the COVID-19 restrictions were introduced. In line with expectations expressed in previous studies (Ertan et al., 2020; Nardi-Rodríguez & Paredes-López, 2022; Sharma & Borah, 2020), neighbors became more important as reporters during the first lockdown. Perhaps the COVID-19 restrictions have led to more bystanders having heard and/or seen domestic violence as people spend more time at home, contributing to greater social control. In addition, our findings reveal that the proportion of non-professional reports is largest during the third identified period in both 2019 and 2020. As this is the period of the Dutch summer holiday, it is likely that people—especially parents with school-going children—were spending time at home more often. These findings further support the idea that domestic violence may be detected and reported more often by bystanders during periods of increased time spent at home.

The increase in the proportion of reports coming from the police in combination with the increase in the proportion of reports coming from neighbors could mean that citizens not only reported their suspicions of domestic violence more often to the police but also found their way to the official domestic violence agencies more often than prior to the COVID-19 pandemic. Directly after the first lockdown was implemented, a national campaign ‘Domestic Violence During the COVID-19 Pandemic’ was launched which was specifically aimed at encouraging bystanders to report suspicions of domestic violence to the authorities. With increasing attention for violent escalations to occur due to restrictions, the willingness among citizens to report domestic violence may therefore also have increased. At the same time, however, the restrictions may have affected the social networks of individuals directly involved in a domestic violence case. As social contact was limited following the restrictions, their social networks could have deteriorated, potentially resulting in fewer opportunities for family members and persons belonging to the social network to detect and report domestic violence among their close relations to the authorities. In the light of these results, changes in the opportunity to report domestic violence could unjustly lead to unchanged levels of reported domestic violence (Anderberg et al., 2020).

As the pandemic continues, future research should address the possibility that the restrictions to control the spread of the COVID-19 virus may have increased pre-pandemic barriers to reporting domestic violence, by examining data from victimization surveys. Fear that perpetrators might find out, for example, is a common reason for victims to not disclose the violence (Rose et al., 2011). The possible constant proximity of the perpetrator following the stay-at-home orders may have exacerbated this fear.

Different authors have argued that it is important to not only examine possible in- and decreases in domestic violence rates but also to address how the COVID-19 crisis has impacted upon the lives of individuals experiencing domestic violence, as well as its impact on support services and frontline workers (Bergman et al., 2021). In this light, we are currently exploring possibilities to get a better understanding of the potential changes in the dark number of domestic violence, and are also gathering more in-depth information by conducting file research and interviews with victims on the circumstances that have led to the violence, which may also specifically identify COVID-19 related escalations, and how victims of domestic violence have evaluated (new forms of) assistance provided by support services.

### Limitations

This study has several important limitations. First, especially at the beginning of 2019, reports of domestic violence were sometimes not recorded in detail. As a result, information about the presumptive nature of domestic violence or the reporters’ role related to the incident was not registered, resulting in many missing values. The time series forecasting models were performed twice to understand if, and how, these missing values affected the outcome of the models by in- and excluding the first ten weeks of 2019, in which most missing values were observed. No differences were found between the two forecasting models for the presumptive nature and the type of reporter of domestic violence.

Second, it would be ideal to include more years of data prior to the COVID-19 pandemic to train the SARIMAX models. However, we could only gather data from the domestic violence agency from 2019 onwards since the 26 different regions only began to register the incoming reports uniformly as of that year.

Third, an important limitation of the official domestic violence records is that the date a report is made does not necessarily reflect the reported violence that took place. Victims of domestic violence could experience thresholds to report the violence (due to shame, feelings of guilt, or out of fear of the consequences when the perpetrator finds out), and therefore it can take a long time before victims decide on whether to report the violence to authorities. We distinguished the four COVID-19 periods and compared them to the same weeks in 2019, but we cannot say with certainty that the violence reported in a specific period also started in these particular periods.

### Conclusion

The empirical findings of this study do not indicate an increase in the official domestic violence records compared to 2019, the year prior to the outbreak of the COVID-19 virus. Our findings do indicate a positive change in the reporting of citizens, especially neighbors. As restrictions are still being up- and downscaled to reduce the spread of the COVID-19 virus, it remains important to keep stimulating citizens, but also victims, their social network, and professionals, to be aware and report their experiences to authorities to prevent continued, if not aggravating, victimization.

## Figures and Tables

**Fig. 7:**
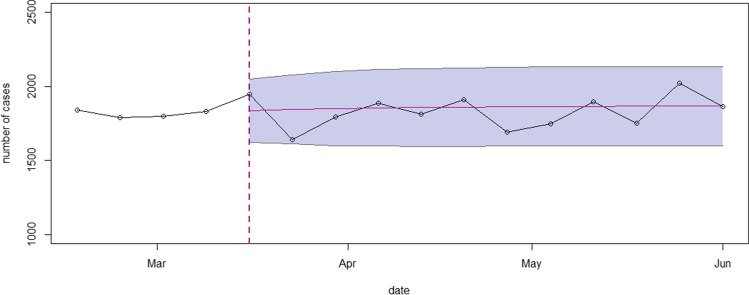
SARIMAX model of the observed and expected number of domestic violence cases per week during the first lockdown. Note: The x-axis represents the forecasted weeks into the first lockdown. The starting date is Sunday March 1st, 2020. The y-axis shows the number of reported and predicted domestic violence cases. Inside the graph, the black line represents the actual number of domestic violence cases per day, the red line is the mean daily prediction, and the confidence interval of the predictions is represented by the blue area around this line. The black dots represent the observed data outside of the prediction confidence interval

**Fig. 8:**
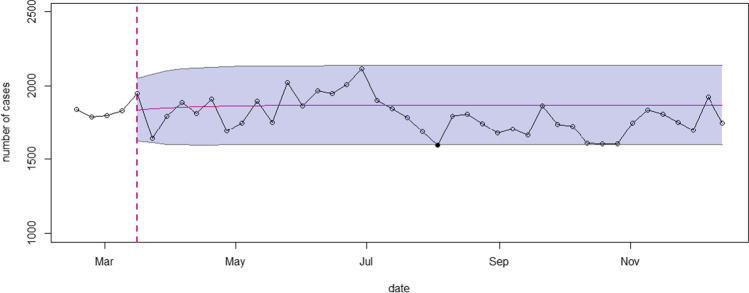
Extended SARIMAX model of the observed and expected number of domestic violence per week during the COVID-19 pandemic. Note: The x-axis represents the forecasted weeks into the COVID-19 pandemic. The starting date is Sunday March 1st, 2020. The y-axis shows the number of reported and predicted domestic violence cases. Inside the graph, the black line represents the actual number of domestic violence cases per day, the red line is the mean daily prediction, and the confidence interval of the predictions is represented by the blue area around this line. The black dots represent the observed data outside of the prediction confidence interval

**Fig. 9:**
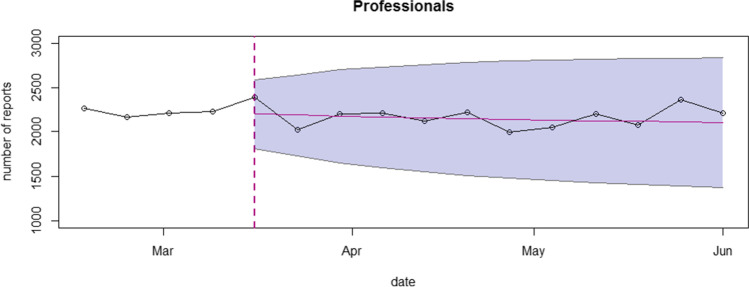
SARIMAX model of the observed and expected number of professional reports per week during the first lockdown. Note: The x-axis represents the forecasted weeks into the first lockdown. The starting date is Sunday March 1st, 2020. The y-axis shows the number of reported and predicted professional reports. Inside the graph, the black line represents the actual number of professional reports per day, the red line is the mean daily prediction, and the confidence interval of the predictions is represented by the blue area around this line. The black dots represent the observed data outside of the prediction confidence interval

**Fig. 10:**
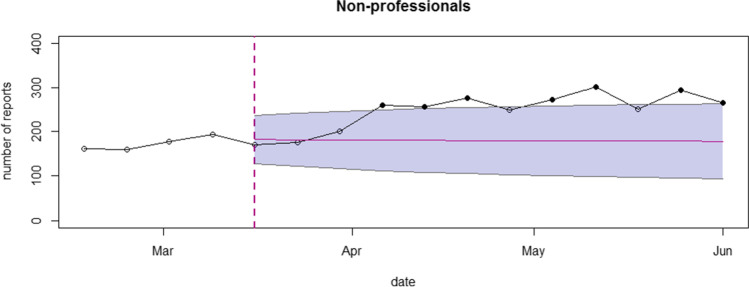
SARIMAX model of the observed and expected number of non-professional reports per week during the first lockdown. Note: The x-axis represents the forecasted weeks into the first lockdown. The starting date is Sunday March 1st, 2020. The y-axis shows the number of reported and predicted non-professional reports. Inside the graph, the black line represents the actual number of non-professional reports per day, the red line is the mean daily prediction, and the confidence interval of the predictions is represented by the blue area around this line. The black dots represent the observed data outside of the prediction confidence interval

## Appendix

The SARIMAX model is a time-series data analysis statistical tool. An ARIMA model is a combination of a non-seasonal Auto-Regressive (AR) and Moving Average (MA) model, using three key time-series parameters: Auto-Regressive term *p* (number of autoregressive terms), order of differencing term *d* (number of times differencing pre-processing step applied to make the time series stationary), and Moving Average term *q* (number of moving average terms). A first inspection of our data revealed a clear weekly pattern in domestic violence cases, i.e., a higher number of cases reported on Mondays, lower number of cases reported during weekends. We therefore chose the SARIMAX modelling approach, because this approach allowed us to include extra parameters and exogenous inputs that account for seasonality, and holidays and day after holidays.

The first step in this approach was to assess whether the time-series was stationary, as required for ARIMA models, by performing an Augmented Dickey Fuller (ADF) test including a seven-period repetitive pattern. Second, we estimated the parameters for non-seasonal and seasonal AR and MA, which are required to be selected beforehand. These parameters were automatically determined by applying the Hyndman – Khandakar algorithm in the fpp3 package in R (Hyndman & Athanasopoulos, 2021; Hyndman & Khandakar, 2008). The algorithm combines unit root tests, minimization of the Akaike Information Criterion (AIC), and Maximum Likelihood Estimation (MLE) to create multiple models with different parameters, ultimately selecting the model with the smallest AIC value. Other approaches, such as Bayesian Structural Time Series (BTST) approach, were performed as a robustness check.

The selected parameters for each of the models are shown in Table 8. Moreover, the mean absolute scale errors (MASE) are included in Table

**Table 8:** Parameters and mean absolute scale errors of SARIMAX models

SARIMAX model	Groups	AR	MA	Seasonal AR	Seasonal MA	MASE
Domestic violence cases	Short forecast	1	3	1	1	0.86
	Long forecast	1	3	1	1	0.99
Cases by nature of domestic violence	IPV and child maltreatment	2	2	1	1	0.65
	Child maltreatment	3	2	0	1	0.74
	IPV	2	2	2	1	0.58
	Violence against parents	0	0	1	1	0.74
	Elderly abuse	1	1	1	1	0.66
	Other problems	5	0	0	1	0.54
Reports by type of reporter	Professionals	1	1	0	1	0.69
	Non-professionals	4	0	0	2	0.23

^8^. A MASE value below 1 indicates that the model performed better than compared to a naïve model. All models performed better than their naïve counterparts﻿.

The final SARIMAX models were used to forecast the predicted trends in domestic violence and their mean square errors (MSE). The MSE is used to calculate the 95% confidence interval. If the observed trends in domestic violence fell outside the upper or lower bounds of the 95% confidence interval on many instances, we concluded that domestic violence trends have changed during the first lockdown. We decided to use daily counts of domestic violence records over weekly counts of domestic violence, because domestic violence reports depend on people’s daily routines. However, since daily counts are more sensitive to noise in the data, we performed the SARIMAX models on a weekly level as a robustness check, see Figures 7–10 and Table

**Table 9:** Plots of weekly SARIMAX models for nature of domestic violence during the first lockdown

	Mismatches	Overestimations	Underestimations
IPV and child maltreatment	5	0	5
Child maltreatment	0	0	0
IPV	1	0	1
Violence against parents	2	0	2
Elderly abuse	1	0	1
Other problems	0	0	0

^9^. Indeed, as compared to the SARIMAX models on a daily level, less observed domestic violence trends appeared to fall outside the upper or lower bounds of the 95% confidence intervals. Still, similar conclusions can be drawn from the SARIMAX models performed on both daily and weekly level. We only found the observed trend of non-professional reports to exceed the confidence interval on substantial instances. The SARIMAX model using weekly counts supported this finding.

Table 8

Table 9

Figure 7

Figure 8

Figure 9

Figure 10

## Footnotes

1 The Veilig Thuis system from which the data were retrieved is dynamic, and, due to delays in registering, additional reports may have been added at a later date.

2 To be able to optimally control for the weekly patterning of domestic violence reports, we created even-sized weeks for 2019 and 2020, starting January 1^st^. Week numbers in our analyses therefore differ slightly from calendar weeks.

3 We performed the SARIMAX models on weekly counts as a robustness check. These results are presented in the Appendix.

4 The findings of the number of reports per case in the first period seem contradictory to the findings for the number of cases and number of reports during the first period, as displayed Fig. 1 and 2. However, this is a consequence of the different measurements used for both analyses. When multiple reports exist around one case in both 2019 and 2020, only the first time the case was reported was counted (thus in 2019) while all reports were counted (thus in both 2019 and 2020).
